# Usutu virus and West Nile virus use a transcellular route of neuroinvasion across an in vitro model of the human blood–brain barrier

**DOI:** 10.1038/s44298-024-00034-4

**Published:** 2024-07-25

**Authors:** Eleanor M. Marshall, Marion Koopmans, Barry Rockx

**Affiliations:** Department of Viroscience, Erasmus Medical Center, Rotterdam, the Netherlands

**Keywords:** Virology, Viral host response, Viral pathogenesis, Virus-host interactions, West nile virus

## Abstract

West Nile virus (WNV) leads to thousands of cases of severe neurological disease in humans each year. Usutu virus (USUV) is closely related to WNV, but rarely induces disease in humans. We hypothesised that USUV is less able to cross the blood-brain barrier (BBB) and, consequently, is less likely to infect the brain. Therefore, we developed an in vitro BBB model consisting of primary human brain microvascular endothelial cells, pericytes and astrocytes. Both USUV and WNV invaded across the in vitro BBB via a transcellular mechanism in the absence of barrier disruption. USUV replicated to lower titres than WNV but induced a comparable cytokine and chemokine response, with modulation of key factors associated with barrier function and immune-cell migration. In conclusion, USUV appears attenuated in its ability to replicate at this interface compared with WNV, but further work must be done to identify key determinants underlying the differing clinical presentations.

## Introduction

Many arthropod-borne viruses (arboviruses) can cause severe neurological disease in humans, but how these viruses bypass the numerous physical and immunological barriers in place to protect the central nervous system (CNS) is not well understood. West Nile virus (WNV) is a mosquito-borne flavivirus that causes thousands of cases of neuroinvasive disease in many regions of the world every year, including North America^1^, and increasingly in Europe^2^, with a continued emergence in new regions each transmission season. Usutu virus (USUV) is phylogenetically closely related to and co-circulates with WNV in Europe, but since the first case of human infection identified in 1981, there have only been around 100 documented cases of USUV-induced disease and no reports of fatal infection^3^. WNV and USUV therefore appear to differ in their ability to cause severe disease in humans, which could stem from many factors, including the ability of the virus to gain access to the CNS via one or more routes of neuroinvasion. Haematogenous routes of neuroinvasion describe the passage of virus from the blood into the brain across physical barriers such as the blood-brain barrier (BBB).

The BBB is a semi-permeable selective border composed of tightly joined brain microvascular endothelial cells (BMECs), ensheathed by pericytes and astrocytes. Cross-talk between these cell types is important for a wide range of essential functions, including barrier permeability and immune responses^4–6^. The BBB is considered an important interface for invasion of viruses into the CNS from the blood. Such invasion can occur via a transcellular route in which the virus must infect or be transported across the cells of the barrier, or via a paracellular route in which virus is able to enter between the cells of a disrupted barrier, either as free virions or within infected immune cells in a so-called Trojan horse mechanism of invasion^7^. For WNV, viraemia in humans is most often transient and low titre, beginning 1–3 days post-infection and persisting until around 6 days post-infection. In a small percentage of infected individuals, there is a rapid onset of neurological disease as viraemia wanes^8^, suggesting passage of virus into the CNS within the viraemic period, followed by subsequent replication, resulting in sufficient damage and inflammation for overt clinical presentation. WNV is thought to use multiple routes of neuroinvasion, including both transcellular and paracellular invasion across the BBB^7^, with both in vivo^9^ and in vitro data showing an initial early passage of virus across an intact barrier^9,10^, followed by disruption of the barrier at later time points^9,11^ allowing for secondary invasion via paracellular mechanisms. Direct in vivo comparison between USUV and WNV is made difficult by the requirement for use of immunocompromised models to allow for development of disease following USUV infection^12^. In vitro, USUV has been shown to invade across a model of “brain-like” endothelial cells with relatively minimal barrier disruption at late time points up to 10 days post-infection^11,13,14^.

However, previous studies investigating the invasion of USUV and WNV across in vitro models of the BBB have used endothelial monocultures^10,15^ from varying organ sources, such as human umbilical cord vein endothelial cells^16^, or employed co-culture with non-human cell types, such as bovine pericytes, in absence of direct cell-cell contact^11,13,14^. Further, human astrocytes, which are a key component of the BBB and produce vasoactive and immunostimulatory factors known to modulate barrier function^5,17^, have as yet been omitted.

Therefore, previous studies have primarily focused on late-stage secondary neuroinvasion or used models that do not recapitulate the origin, components nor direct interaction of the cells of the human BBB. A direct comparison of the early replication and invasion kinetics of USUV and WNV across a more biologically relevant model of the human BBB, and the relative immune responses induced at these early time points, is yet to be carried out. Here, we aimed to determine whether USUV and WNV differ in their capacity to invade the CNS across the BBB by employing a triple-coculture in vitro transwell system, using primary human BMECs, astrocytes and pericytes in a physically proximate culture, to recapitulate the cell-cell interactions of the human BBB and investigate the mechanism of early viral invasion across this barrier. Identifying key differences and similarities between these two viruses will shed light on the essential virus or host factors that underlie viral neuroinvasion, and thereby aid in assessment of the future risk posed by USUV. We also provide a robust, biologically relevant platform that can be employed to evaluate the neuroinvasive capacity of emerging viruses and live-attenuated vaccine candidates, and to develop therapeutic strategies working to prevent viral neuroinvasion across the BBB.

## Results

### USUV and WNV can infect and replicate within all three cell types of the human blood-brain barrier

**Fig. 1:**
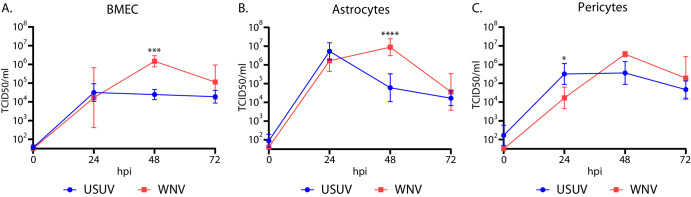
Usutu virus and West Nile virus can replicate in all cell types of the human blood-brain barrier. Replication kinetics of primary human (**A**) Brain microvascular endothelial cells (BMECs), (**B**) astrocytes and (**C**) pericytes infected with Usutu virus (USUV) and West Nile virus (WNV) at a multiplicity of infection of 1. *n* = 2. 3 replicates per condition, per experiment. *p = 0.0158. ***p = 0.0006. ****p < 0.0001. Data displayed has been log-transformed (Y = log[Y]). 2-way ANOVA with multiple comparisons carried out on log-transformed data.

To identify the tropism of USUV and WNV for the different cell types of the human BBB we infected primary human BMECs, astrocytes and pericytes with USUV or WNV at an MOI of 1. Both USUV and WNV could infect and replicate in BMECs (Fig. 1A), with WNV peaking at a mean titre of 1.47 x 10^6^ TCID50/ml at 48 hpi and showing a significantly higher (*p* = 0.0006) titre over USUV at this time point. WNV titres decreased by 72 hpi to 1.14 x 10^5^ TCID50/ml, and did not show a significant difference to USUV at this time point. Similar kinetics were observed in primary human astrocytes (Fig. 1B), with comparable titres for both viruses at 24 hpi, leading to a significantly higher (*p* < 0.0001) mean WNV titre of 8.80 x 10^6^ TCDI50/ml when compared with USUV, which reached 6.00 x 10^4^ TCID50/ml at 48 hpi. Both USUV and WNV showed a decrease in mean titres at 72 hpi to 1.67 x 10^4^ TCID50/ml and 3.60 x 10^4^ TCID50/ml respectively. In pericytes (Fig. 1C), USUV had a significantly higher (*p* = 0.0158) titre of 3.16 x 10^5^ TCID50/ml compared with 1.67 x 10^4^ TCID50/ml for WNV at 24 hpi. However, whilst USUV plateaued after 24 hpi, WNV titres continued to increase, peaking at a mean titre of 3.59 x 10^6^ TCID50/ml at 48 hpi. We did not observe overt cytopathic effects (CPE) in any cell type for either virus.

**Fig. 2:**
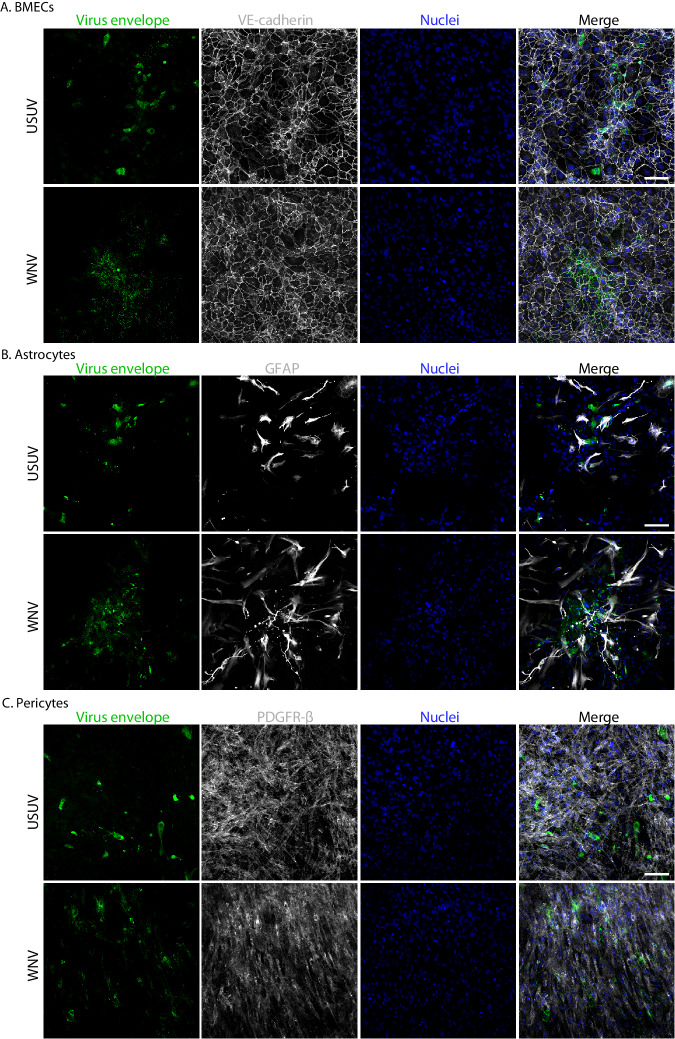
Usutu virus and West Nile virus infection can be visualised in all cell types of the human blood-brain barrier by immunofluorescent staining. **A** Immunofluorescent (IF) staining of Usutu virus- (USUV) and West Nile virus- (WNV) infected brain microvascular endothelial cells (BMECs). VE-cadherin is shown in white. **B** IF staining of USUV- and WNV-infected astrocytes. GFAP is shown in white. **C** IF staining of USUV- and WNV-infected pericytes. PDGFR-β is shown in white. Nuclei are shown in blue. The virus envelope is shown in green. Scale bars represent 100 μm.

We then carried out immunofluorescent (IF) staining to confirm infection within the primary cell cultures, in combination with relevant cell markers. We identified staining for viral envelope of USUV and WNV in scattered foci throughout BMEC (Fig. 2A), astrocyte (Fig. 2B) and pericyte (Fig. 2C) cultures, and all three cultures stained for their expected markers. Overall, we found that all three cell types of the human BBB are susceptible to infection with USUV and WNV.

### The triple-coculture in vitro blood-brain barrier exhibits the greatest barrier function

**Fig. 3:**
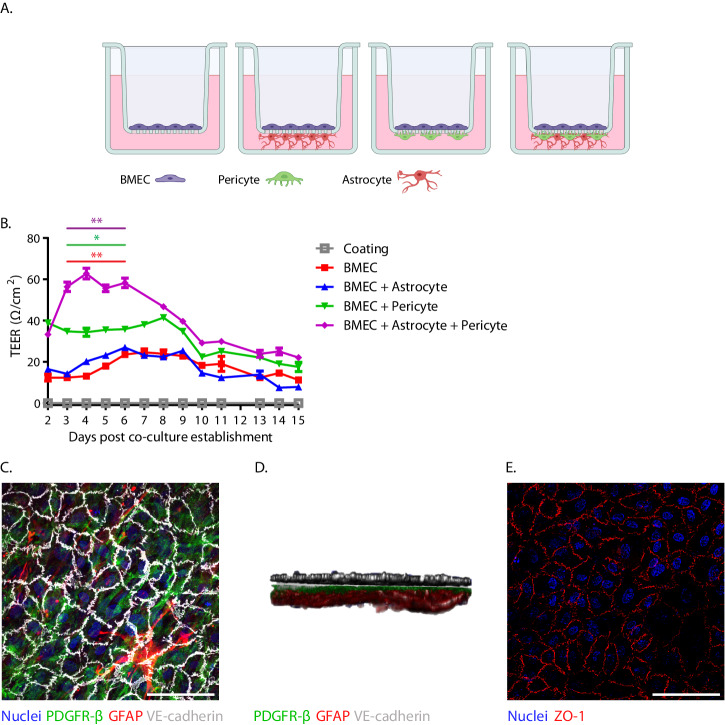
A triple-coculture of brain microvascular endothelial cells, astrocytes and pericytes provides the highest barrier function. **A** Representation of the four different blood-brain barrier (BBB) culture set-ups, with brain microvascular endothelial cells (BMECs) in the apical compartment and astrocytes, pericytes or astrocytes and pericytes seeded on the basolateral membrane of the transwell. Made using biorender. **B** Transendothelial resistance (TEER) of the BBB culture set-ups following culture establishment. Three replicates per condition. Representative data from three independent experiments. Purple stars indicate BMEC monoculture vs BMEC + pericyte + astrocyte triple-coculture (**p < 0.0051). Green stars indicate BMEC + pericyte double-coculture vs BMEC + pericyte + astrocyte triple-coculture (*p < 0.0136). Red stars indicate BMEC monoculture vs BMEC + pericyte double-coculture (**p < 0.0078). 2-way ANOVA with multiple comparison run on stated conditions for day 3–day 6 post coculture establishment. To aid in visualisation, the *p* value stated and presented is the least significant value obtained from the multi-time point comparison. **C** XY view of immunofluorescent (IF) stained triple-coculture in vitro BBB viewed from the apical side. PDGFR-β is shown in green. GFAP is shown in red. VE-cadherin is shown in white. **D** XZ view of 3D rendered, IF stained triple-coculture in vitro BBB. **E** IF staining of ZO-1 in BMECs of apical compartment. Nuclei are shown in blue. ZO-1 is shown in red. Scale bars represent 100 μm.

Next, to confirm data previously obtained in a similarly proximate coculture model^18^, we investigated to what extent coculturing the primary astrocytes and pericytes with BMECs contributed to barrier establishment. We seeded BMECs in the apical compartment alone, or in combination with either astrocytes, pericytes or both of these cell types seeded on the basolateral side of the transwell membrane (Fig. 3A). In the absence of pericytes, astrocytes did not contribute to the barrier function, with the BMEC + astrocyte coculture exhibiting low transendothelial electrical resistance (TEER) values similar to the monoculture of BMECs. The barrier of the BMEC monoculture peaked on day 7 post-culture establishment at 24.64 Ω/cm^2^ and the BMEC + astrocyte cocultures peaked on day 6 at 26.88 Ω/cm^2^. The addition of pericytes did contribute to the barrier, with the BMEC + pericyte coculture showing persistently higher TEER compared with the BMEC alone until day 8 where it peaked at 41.44 Ω/cm^2^. The BMEC, astrocyte, pericyte triple-coculture showed the highest TEER, which rapidly increased between day 2 to 3, then peaked at day 4 at 62.72 Ω/cm^2^ and stayed >2 fold higher than BMEC alone until day 6 post-seeding when the triple-coculture dropped to TEER values similar to BMEC + pericytes (Fig. 3B). IF staining of the in vitro BBB confirmed the presence of all three cell types (Fig. 3C) with 3D rendering revealing the expected orientation in relation to the transwell membrane (Fig. 3D, Vid. S1). In addition to assessment of the barrier using TEER measurement, we also carried out IF staining of the BMECs in the apical compartment for the key tight-junction protein, ZO-1, and found that ZO-1 was expressed in our in vitro BBB system (Fig. 3E). These data suggest that coculturing primary BMECs, astrocytes and pericytes has a synergistic effect on BBB barrier function.

### USUV and WNV can invade across the in vitro blood-brain barrier in absence of barrier disruption

To investigate early events of viral invasion, we initially performed a pilot study with WNV to determine on what timescale initial invasion occurred. We found that viral genome could not be detected in the basolateral compartment until around 16 hpi, assumedly after a first round of replication in BMECs (Fig. S1A–D). This lag was not caused by a blockade of virion diffusion by the coated transwell membrane (Fig. S2).

**Fig. 4:**
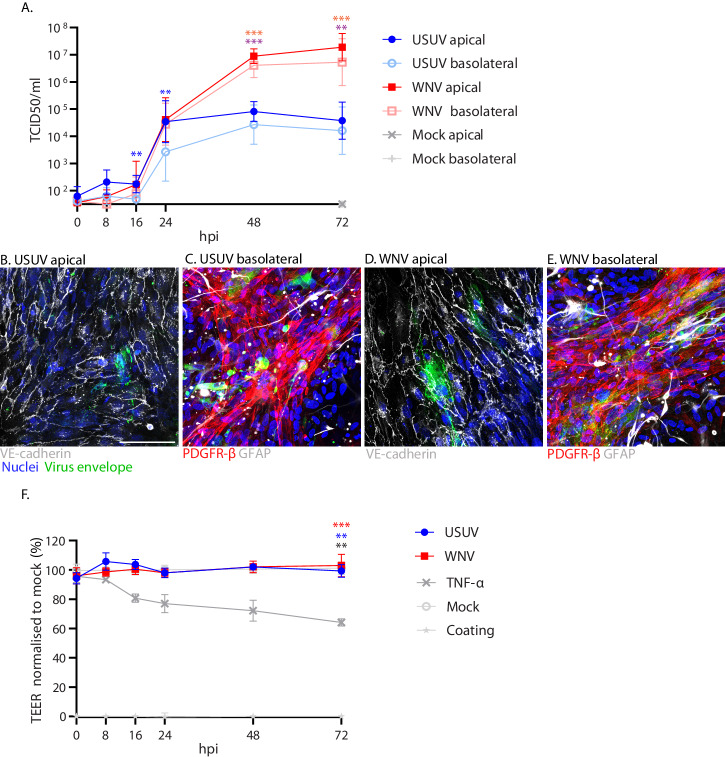
Usutu virus and West Nile virus infect and invade across the in vitro blood-brain barrier within the same time frame and do not cause barrier disruption. **A**. Growth kinetics in the apical and basolateral compartments of Usutu virus- (USUV) and West Nile virus- (WNV) infected in vitro blood-brain barriers (BBB), infected at a multiplicity of infection of 1. *n* = 2. Three replicates per condition, per experiment. Mean with SD. Blue stars indicate USUV apical vs USUV basolateral. 16 hpi **p = 0.0089. 24hpi **p = 0.003. Purple stars indicate USUV apical vs WNV apical. ***p = 0.0005. Orange stars indicate USUV basolateral vs WNV basolateral. **p = 0.0029. ***p = 0.0008. Data displayed has been log-transformed (Y = log[Y]). 2-way ANOVA with multiple comparison carried out on log-transformed data. **B** Immunofluorescent (IF) staining of USUV-infected brain microvascular endothelial cells (BMECs) in apical compartment (VE-cadherin shown in white) and (**C**) astrocytes and pericytes in the basolateral compartment of the in vitro BBB at 72 hpi. Nuclei are shown in blue. USUV envelope protein shown in green. PDGFR-β is shown in red. GFAP is shown in white. **D** IF staining of WNV-infected BMECs in apical compartment and (**E**) astrocytes and pericytes in the basolateral compartment of the in vitro BBB at 72 hpi. Representative images from two independent experiments. Scale bars represent 100 μm. **F** Transendothelial electrical resistance (TEER) of USUV- and WNV-infected and TNF-α stimulated in vitro BBBs across the course of the growth-kinetics experiment. *n* = 2. Three replicates per condition, per experiment. Red stars indicate TNF-α vs WNV. Blue stars indicate TNF-α vs USUV. Grey stars indicate TNF-α vs mock. **p = 0.0016. *** *p* = 0.0003. 2-way ANOVA with multiple comparisons.

Based upon this data, we chose both 16 hpi and 24 hpi time points to compare the kinetics of invasion for USUV and WNV, and also investigated whether USUV and WNV impact the barrier function on a longer timescale, following the initial invasion. As expected, WNV could not be detected in the basolateral compartment until the 16 hpi time point (Fig. 4A). USUV showed a similar pattern of invasion, increasing rapidly between 16 hpi and 24 hpi from 4.84 x 10^1^ TCID50/ml to 2.67 x 10^3^ TCID50/ml in the basolateral compartment. Titres in the apical compartment were comparable between USUV and WNV at 16 and 24 hpi. USUV titres in the apical compartment peaked at 8.08 × 10^4^ TCID50/ml at 48 hpi but WNV continued to increase to significantly higher (*p* = 0.0005) titres compared with USUV at 48 hpi and 72 hpi, reaching a titre of 1.89 × 10^7^ TCID50/ml. The titres in the basolateral compartments of each virus-infected condition showed similar kinetics to the apical compartments. WNV showed no significant difference between the apical and basolateral compartments at any time point. However, at 16 hpi (*p* = 0.0089) and 24 hpi (*p* = 0.003) USUV did show a significant difference, with lower titres in the basolateral versus the apical compartment, but by 48 hpi USUV titres were comparable in both compartments. We confirmed infection of cells in both the apical and basolateral compartments with USUV (Fig. 4B, C) and WNV (Fig. 4D, E) via IF staining of viral envelope and respective cell markers of each compartment. All three cell types of the in vitro BBB were infected at 72 hpi in both virus conditions. This infection did not result in significant changes in TEER compared to mock at any time point for either virus, whilst addition of TNF-α, as a positive control, led to clear disruption of barrier function to a mean of 64% of the mock-infected control at 72 hpi (Fig. 4F). These data show that USUV invaded across the in vitro BBB within the same timescale as WNV, and that neither virus-induced disruption of the barrier following this initial invasion.

### WNV and USUV infection of the in vitro blood-brain barrier induces changes in key factors associated with barrier function, anti-viral immunity and immune cell transmigration

**Fig. 5:**
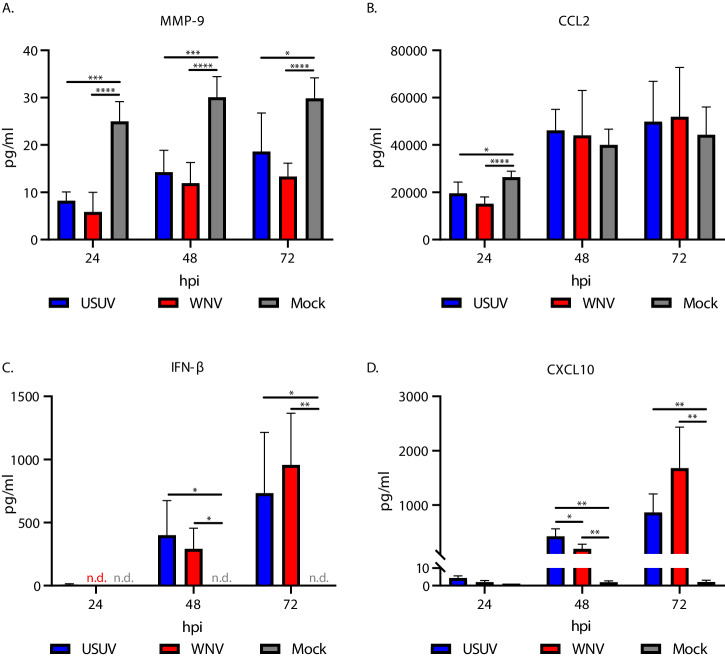
Usutu virus and West Nile virus infection of the in vitro blood-brain barrier induces changes in key factors associated with barrier function, anti-viral immunity and immune cell transmigration. Concentrations of (**A**) MMP-9 (*p = 0.0435. **** *p* < 0.0001. 24hpi *** *p* = 0.0001. 48 hpi *** *p* = 0.0003) **B** CCL2 (* *p* = 0.0368. **** *p* < 0.0001) **C** IFN-β (48hpi * Usutu virus (USUV) vs mock *p* = 0.0365, West Nile virus (WNV) vs mock *p* = 0.0162. 72 hpi * *p* = 0.0306, **p = 0.0053) and **D** CXCL10 (* *p* = 0.0225. 48hpi ** USUV vs mock *p* = 0.0018, WNV vs mock *p* = 0.006. 72 hpi ** USUV vs mock *p* = 0.0037, WNV vs mock *p* = 0.0068.) in basolateral supernatants from in vitro BBBs infected at MOI 1 with WNV or USUV. 2-way ANOVA with multiple comparison. *n* = 2. Three replicates per condition, per experiment. Mean with SD.

To characterise the host response mounted upon USUV and WNV infection of the in vitro BBB, we identified the concentration of a range of cytokines and chemokines associated with barrier disruption and immune response to viral infection in the supernatants of infected and mock-infected in vitro BBBs (Fig. 5A–D. Fig. S3A–L).

As we observed no disruption of barrier function in the infected conditions, we hypothesised that there would be little difference between infected and mock-infected conditions in the expression of factors known to directly increase permeability of the BBB. USUV- and WNV-infected conditions showed a significantly reduced concentration of MMP-9 at all time points compared with mock (Fig. 5A) which was most apparent at 24 hpi. At this time point, mock infected conditions showed a mean of 24.98 pg/ml of MMP-9, whereas USUV and WNV had concentrations of 8.21 pg/ml (*p* = 0.0001) and 5.86 pg/ml (*p* < 0.0001) respectively. MMP-3 was not down-regulated in infected conditions, with both USUV and WNV having comparable concentrations to the mock-infected conditions at 24 hpi and 48 hpi, and increased concentrations at 72 hpi which was significant for WNV (*p* = 0.0043) with a mean MMP-3 concentration of 24.10 pg/ml compared to 16.04 pg/ml for mock (Fig. S3J). Additionally, we found that CCL2 was highly expressed in the in vitro BBB, with concentrations in mock peaking at 4.43 x 10^4^ pg/ml at 72 hpi, but infection with USUV (*p* = 0.0368) and WNV (*p* < 0.0001) resulted in a significantly lower concentration at 24 hpi compared to mock (Fig. 5B).

Whilst concentrations of MMP-9 and CCL2 were reduced upon infection, IFN-β (Fig. 5C) and CXCL10 (Fig. 5D) were induced as the infection course progressed, with significant increases over mock after 48 hpi and 72 hpi for both the USUV- and WNV-infected conditions. At 48 hpi, USUV induced a significantly increased (*p* = 0.0225) expression of CXCL10 compared with WNV, with concentrations of 4.22 x 10^2^ pg/ml and 1.94 x 10^2^ pg/ml respectively, but this difference between viruses was no longer significant at 72 hpi. Overall, these data show reduction of MMP-9 and CCL2 concentrations, and an induction of IFN-β and CXCL10 across time, as a result of infection.

## Discussion

WNV is thought to employ both transcellular and paracellular modes of invasion across the physical barriers that separate the blood from the CNS, such as the BBB. The BBB acts as a key entry point for neuroinvasion, but the ability of USUV to invade the CNS across this barrier is not well understood^7^. Here we show that all three cell types of the human BBB are susceptible to infection with USUV and WNV. Previous work has shown susceptibility of human endothelial cells, pericytes and astrocytes to infection with USUV after four days of infection^13^, but the kinetics of USUV replication in these cells, combined with a direct comparison with WNV, has not been carried out. We found USUV and WNV demonstrated similar replication kinetics, with comparable titres observed at 72 hpi between USUV and WNV in all three cell types, despite WNV peaking higher and later than USUV in BMECs and astrocytes. This contrasts with the data obtained from our in vitro BBB, in which WNV grew to significantly higher titres than USUV after 24 hpi. Prior to 24 hpi, the kinetics of invasion were comparable between USUV and WNV, and the absence of barrier disruption at any time point indicates a transcellular mode of invasion. A previously published in vitro human umbilical vein endothelial cell model, co-cultured with bovine pericytes, showed similar results for USUV^14^, however WNV was found to induce severe endothelial barrier impairment at late time points and replicated less efficiently than USUV^11^, thereby contrasting with our data. Yet, a study similarly investigating invasion up to 3 days post-infection did not observe WNV to induce barrier disruption in a BMEC monoculture barrier^10^. Whilst variation exists in the MOIs and viral strains used, an important difference and novelty of our work compared with earlier data is our use of primary human BMECs, astrocytes and pericytes in a physically proximate triple-coculture. Unlike models previously employed^10,11,13–16^, the origin, orientation and interaction of cells within this triple-coculture in vitro BBB provide a more physiologically relevant model for the study of invasion of USUV and WNV at this interface, as evidenced by the synergistic effect on the barrier function of the BBB.

Additionally, we primarily focused on the early phase of initial invasion across the BBB, between 0 and 72 hpi, not later stage effects of viral replication and secondary invasion, up to 10 dpi as has already been studied^11^. Further, our in vitro data is in line with previously published in vivo data, describing an initial entry of WNV into the brain in absence of BBB disruption^9^ followed by a later disruption due to infection of neural cells, leading to infiltration of immune cells that contribute to barrier disruption via release of chemotactic and inflammatory factors^7,19,20^.

Previously, elevated levels of MMP-9 have been implicated in contributing to BBB disruption during WNV infection^17,21^, but in our in vitro BBB we observed a significant decrease in MMP-9, but not MMP-3, concentration in both USUV- and WNV-infected conditions. MMP-9 is involved in the degradation and remodelling of components of the extracellular matrix and is essential for a wide variety of physiological processes, including angiogenesis and cell migration. MMP-3 is also involved in extracellular matrix degradation but additionally functions to activate other MMPs, including MMP-9, via proteolytic cleavage of MMP pro-peptides^22^. MMP expression is tightly regulated at all levels of protein production, including by action of tissue inhibitors of MMP (TIMP) which show relatively specific interactions with pro-MMPs^23^. TIMPs are known to be expressed by all three cell types of the BBB^24^ and induction of TIMP expression in response to infection has been shown for WNV and yellow fever virus^25^, as well as for other viruses, including Japanese encephalitis virus, respiratory syncytial virus^26^, human cytomegalovirus^27^ and influenza virus^28^. However, other studies have shown a down-regulation of TIMPs and an upregulation of MMPs in response to WNV^17^. These contradictions may stem from the use of different cell types and investigation at different time points post-infection. Future work could aim to identify the relative TIMP/MMP balance within the triple-coculture BBB throughout the infection course, to elucidate the mechanism underlying the reduction in the secretion of MMP-9, but not MMP-3, as a result of infection.

CCL2 has also been found to increase endothelial permeability^29,30^. However, we observed a reduction in the concentration of CCL2 in the USUV- and WNV-infected conditions compared to mock at 24 hpi. As both WNV and USUV-infected in vitro BBBs showed comparable measures of barrier function to mock, the reduction in MMP-9 and CCL2 concentrations was therefore not sufficient to induce a direct, detectable decrease in permeability of the barrier as a response to infection. The implications and underlying mechanisms of our observed reduction of MMP-9 and CCL2 upon USUV and WNV infection require further elucidation.

Future work with the in vitro BBB model employed in this study should include addition of in vitro or ex vivo brain cultures in the basolateral compartment, thereby modelling the entire process of neuroinvasion and subsequent infection of the brain, and increasing the relevance of studying secondary neuroinvasion and barrier disruption of the BBB at later time points. For the study of secondary invasion, the 4–5 day persistence of barrier function in the triple-coculture model acts as an upper limit for the time scale of experiments. However, as we found the initial invasion of the virus to occur within 24 hpi, there would still be sufficient time for numerous rounds of replication within the basolateral compartment remaining.

Reproducible experimental USUV infection of the CNS can only be achieved in vivo using neonatal mouse models, in which the BBB is not yet fully formed^31^, or in severely immune-compromised mouse models^12^. Contrastingly, WNV can induce neurological disease in immunocompetent mice^7,32^, indicating that physical or immune barriers prevent neuroinvasion of USUV. In the in vitro BBB, WNV reached significantly higher titres in both compartments compared to USUV. These data suggest a more effective control of USUV replication at early time points by the anti-viral response. USUV has previously been found to have an increased sensitivity to type I and type III interferon (IFN) responses, compared with WNV^33^, and the resistance of WNV to type I IFN signalling was found to be a key determinant in its replication fitness and virulence^34^. We saw few significant differences between USUV and WNV in the cytokine profiles of their respective basolateral supernatants, despite WNV growing to significantly higher titres, suggesting a dampened response to infection with WNV. As the BMECs in the apical compartment did not release virus into the basolateral compartment until around 16 hpi, the exposure of the astrocytes and pericytes in the basolateral compartment to virus in the supernatant was delayed until this point. This may explain the relatively low presence of some of the cytokines we investigated at 24 hpi, including IFN-β and CXCL10, that may be dependent on infection or activation of astrocytes or pericytes^7,35,36^.

Ultimately, the anti-viral response was activated in the infected conditions, with increasing IFN-β concentrations following the 24 hpi time point. CXCL10 is an IFN-induced chemokine^37^ that promotes extravasation and activation of CXCR3 positive immune cells^38^, and has been shown to play an important role in protection of the CNS during WNV infection^39,40^. In our USUV- and WNV-infected in vitro BBBs, we saw significant induction of CXCL10 in line with increasing IFN-β concentrations. Homing of immune cells towards the site of an infection plays a paradoxical role in neuroinvasive disease, on one hand, aiding in immune control and clearance of the virus, on the other, contributing to immunopathology and potentially exacerbating viral invasion via the Trojan horse mechanism. Invasion via Trojan horse has already been suggested for WNV^7^, however this has not been well studied in vitro and the potential for USUV to employ this route is unknown. Monocytes and dendritic cells have been shown to be permissive to USUV infection, and showed increased binding to an uninfected in vitro ‘brain-like’ endothelial barrier model, whereas non-infected immune cells showed increased binding to an infected barrier at 7 dpi^11^. As we have identified that infection with USUV and WNV leads to early modulation of factors associated with attraction toward and migration across the BBB by host-immune cells, follow-up studies will focus on combination of our in vitro BBB with primary human immune cells to further investigate the mechanisms of (infected) leucocyte extravasation across an (infected) human BBB.

In conclusion, in this study we aimed to identify the capacity of USUV to employ haematogenous routes of neuroinvasion and compare with WNV to gain an insight into potential mechanisms underlying the differing clinical presentation and severity resulting from infection with these viruses. We have shown that both USUV and WNV can use a transcellular mode of invasion in the absence of barrier disruption. The attenuation in replicative capacity of USUV within the BBB may contribute to the observed reduction in severity and frequency of human disease, compared with WNV. However, we have shown that, when given access to the BBB, USUV is able to invade. Further work must be done to investigate the capacity of USUV to employ alternate routes of neuroinvasion and identify key factors underlying the disparate disease severity resulting from infection with USUV versus WNV.

## Methods

### Virus strains and culturing

All viruses were grown and passaged on Vero cells (African green monkey kidney epithelial cells, ATCC CCL-81). Cells were infected at a multiplicity of infection (MOI) of 0.01 and incubated at 37 ^o^C for 5–6 days in Dulbecco’s modified Eagle’s medium (DMEM; Lonza) with 2% FBS (Sigma-Aldrich), 100 U/ml penicillin, 100 μg/ml streptomycin (Lonza), 1% sodium bicarbonate (Lonza) and 2mM L-glutamine (Lonza). Supernatant was harvested, spun down at 4000 *g* for 10 min and aliquoted then frozen at −80 ^o^C. The virus strains used in this study were selected based upon their circulation in the Netherlands and included: USUV (lineage Africa 3, GenBank accession MH891847.1, EVAg 011V-02153, isolated in 2016 from *Turdus merula*) and WNV (lineage 2, GenBank accession OP762595.1, EVAg 010V-04311, isolated in 2020 from *Phylloscopus collybita*). Both viruses were used at passage 3, and were sequenced to ensure no introduction of mutations leading to amino-acid changes compared with the original isolate.

### Cell culture

For human astrocytes (HA, Sciencell) and human pericytes (HP, Sciencell), flasks were coated with 2 μg/cm^2^ poly-L-Lysine (Sciencell) diluted in de-ionised, sterile water and incubated at 37 °C, 5% CO_2_ for a minimum of 1 h to a maximum of 24 h. Flasks were washed twice with de-ionised water. For human brain microvascular endothelial cells (BMECs, Cell systems) flasks were coated with 2–5 ml of pre-warmed 1% gelatin (Sigma Aldrich) dissolved in PBS and incubated at 37 °C, 5% CO_2_ for a minimum of 15 min to a maximum of 24 h.

Sub-confluent cell flasks were washed with PBS before the addition of 0.25% Trypsin-EDTA (Gibco) and incubation at 37 °C, 5% CO_2_ for 2-5 mins. Trypsin was inactivated with 5 ml of FBS and 5 ml of cell-type specific culture medium (Astrocyte medium; AM, Sciencell. Pericyte medium; PM, Sciencell. BMEC medium; MV2, Promocell). Cell suspensions were spun at 120 g for 5 min and resuspended in their respective medium prior to passage into a pre-coated flask. HP were used between passages 3 and 10. HA were used between passages 4 and 10. BMECs were used between passages 7 and 12.

### Transwell seeding

ThinCert™ (Greiner) 12-well, translucent inserts with 3 µm pores were used throughout the study. On day 1, the basolateral sides of the membrane inserts were coated by turning inserts upside-down so the basolateral side faced upward. 100 μl of 2 μg/cm^2^ poly-L-Lysine was pipetted onto the basolateral membrane and spread evenly with the side of a 200 μl pipette tip, before incubating for a minimum of 1 h at 37 °C, 5% CO_2_. Coating solution was then removed and the plate containing inserts was returned to the normal orientation. The inserts were washed twice with de-ionised water and then were inverted and left to dry in the biosafety cabinet for 1 h. 100 μl of 3 × 10^6^/ml HA cell suspension was pipetted onto the basolateral membrane and incubated for 2–3 h at 37 °C, 5% CO_2_. Transwells were reverted and 1100 μl of HA medium was added to the basolateral and 800 μl was added to the apical compartment. On day 2, all medium was removed and transwells were inverted. 100 μl of 6 × 10^5^/ml HP cell suspension was pipetted onto the basolateral membrane and incubated for 2–3 h at 37 °C, 5% CO_2_. The transwells were then reverted and 1100 μl of a 1:1 mix of HA and HP medium was added to the basolateral and 800 μl was added to the apical compartment. On day 4, all medium was removed from the apical compartment and replaced with 150 μl of 1% gelatin solution and then incubated for 1 h at 37 °C, 5% CO_2_. Gelatin solution was removed and 150 μl of 7.5 × 10^5^/ml BMEC cell suspension was pipetted into the apical compartment and incubated for 4–6 h at 37 °C, 5% CO_2_. The basolateral medium was then replaced with 1200 μl of a 1:1 mix of HA and HP medium and 800 μl of MV2 medium was added to the apical compartment.

### Transendothelial electrical resistance assay

An EVOM2 trans-endothelial electrical resistance (TEER) metre with STX2 chopstick electrodes (World Precision instruments) was used to assess barrier function. TEER of a poly-L-lysine and gelatin-coated transwell was used as a coating-only control. Electrodes were first sterilised in 70% ethanol for 5 min, then moved to deionised water, PBS and finally a 1:1 mix of AM/PM medium. Electrodes were then placed into the apical and basolateral compartments of the transwell inserts. TEER was measured and normalised to the coating-only control and surface area of the transwell to obtain normalised Ω/cm^2^.

### Infection of in vitro blood-brain barrier cells

For infection of the monocultured cell types, 48-well plates were coated for 1 h with 2 μg/cm^2^ poly-L-Lysine, or for 15 min with 1% gelatin. HA (2.5 × 10^5^ cells/well), HP (2 × 10^5^ cells/well) and BMEC (1 × 10^5^ cells/well) were seeded in a 48-well plate in their respective medium and used for infection experiments the following day. In vitro BBB cultures were used for infection experiments after 4 days post-BMEC seeding. Barrier function of the BBB cultures was confirmed via TEER prior to infection. For infection of BBB cultures, all medium in the apical compartment was removed before addition of virus inoculum diluted in MV2 medium to an MOI of 1, calculated based on the number of BMECs. MV2 medium alone was used for the mock-infected condition. For infection of monocultures, virus inoculum diluted to an MOI of 1 in the cell-type specific medium was used for infection. Plates were returned to the incubator at 37 °C for 1 h then virus inoculum was removed and the well, in case of monoculture infection, or apical compartment, in case of BBB culture infection, was washed three times with PBS before addition of cell-type specific medium. For the BBB cultures, sample was taken from both the apical and basolateral compartments for time point 0 hpi, and transwells were then transferred to a clean plate with fresh AM/PM basolateral medium. Plates were returned to the incubator at 37 °C. When stated, as a positive control for barrier disruption, uninfected cultures were treated with 100 ng/ml of TNF-α in the apical compartment, which was maintained throughout the experimental course. The supernatant was removed and refreshed at the specified time points. The harvested supernatants were stored at −80 °C for titration or RNA isolation.

### Virus titration

Tenfold serial dilutions of culture supernatants were inoculated onto a semiconfluent monolayer of Vero cells in a 96-well plate (2.3 × 10^4^ cells/well). CPE was used as titre read out and determined at 6 days post-infection (dpi). Virus titres were calculated as the 50% tissue culture infective dose (TCID50) using the Spearman-Kärber method^41^. An initial 1:10 dilution of supernatant resulted in a detection limit of 31.6 TCID50/ml.

### RNA isolation and real-time reverse transcription quantitative PCR for quantification of virus

Sample supernatants in MagnaPure lysis buffer were incubated with Agencourt AMPure-XP (Beckman Coulter) magnetic beads in a 96-well plate. The plate was placed on a DynaMagTM-96 magnetic block (Invitrogen) and supernatant was removed. The beads were washed three times with 70% ethanol whilst on the magnetic block and then left to air dry. The plate was removed from the block and beads were resuspended in de-ionised water to elute the isolated RNA.

A real-time TaqMan^TM^ assay was performed using the Applied-Biosystems 7500 real-time PCR system (ThermoFisher Scientific). WNV primer/probe mix (Forward-primer sequence: CCACCGGAAGTTGAGTAGACG, Reverse-primer sequence: TTTGGTCACCCAGTCCTCCT, Probe sequence: TGCTGCTGCCTGCGGCTCAACCC) was diluted in TaqManTM fast virus 1-Step Master Mix and de-ionised water to a final volume of 12 µl before addition of 8 µl sample RNA. The following programme was used: 5 min 50 °C, 20 s 95 °C and 45 cycles of 3 s 95 °C and 30 s 60 °C. Samples were compared to a standard curve of virus stock dilutions to acquire a TCID50 equivalent/ml.

### Dextran permeability assay

20 kDa fluorescent TRITC dextran (Sigma Aldrich) was added to the apical compartment of the in vitro BBB at 12 hpi to a final concentration of 100 µg/ml, and incubated until 24 hpi. Fluorescence of the basolateral supernatant was assessed using a Tecan Infinite F200 Pro. Data were normalised to fluorescence in the mock-infected condition. BBB cultures treated with 100 ng/ml of TNF-α were used as a positive control for barrier disruption.

### Multiplex immunoassay

To determine concentrations of a panel of human cytokines and chemokines in cell culture supernatants, a custom human magnetic Luminex screening assay, 16-plex kit (R&D systems) was used according to the manufacturer’s instructions. This assay detects target proteins in the cell supernatant via binding to colour-coded beads that have been pre-coated with analyte-specific capture antibodies. Measured proteins were: CCL2, CXCL10, G-CSF, GM-CSF, ICAM-1, IFN-α, IFN-β, IL-1β, IL-6, IL-8, IFN-lambda 2, IFN-lambda 3, MMP-3, MMP-9, TNF-α and VCAM-1. Samples were read using a Bio-Plex suspension array system with automated concentration calculation based upon relation to standard curves.

### Immunofluorescent staining

Cells were fixed for 30 min in 10% formalin. Fixed cells were permeabilised with 0.5% triton (Sigma) diluted in PBS for 15 min, then blocked with 5% bovine serum albumin (BSA; Aurion) for 1 h before incubation with primary-antibodies diluted in PBS with 2% BSA overnight at 4 °C. Cells were washed three times in PBS then incubated with secondary-antibodies for 1 h at room temperature (RT) in the dark. Cells were washed and then incubated with Hoechst (1:1000, Invitrogen) for 20 min at RT in the dark. Images were obtained using a Zeiss LSM 700 laser scanning microscope. Primary antibodies used in this study were: mouse anti-flavivirus envelope protein (1:250, D1-4G2-4–15 hybridoma; ATCC, USA), goat anti-VE-Cadherin (1:100, R&D systems), rabbit anti-PDGFRβ (1:200, clone Y92, Abcam), rabbit anti-GFAP (1:200, Millipore), chicken anti-GFAP (1:200, Abcam). An AF555 conjugated mouse antibody was used to stain ZO-1 (1:100, Thermofisher Scientific) followed by incubation with a secondary antibody to amplify the fluorescent signal. The secondary antibodies (Invitrogen) used in this study were: anti-mouse AF488 (1:250), anti-rabbit AF488 (1:200), anti-mouse AF555 (1:200), anti-rabbit AF555 (1:200), anti-chicken AF555 (1:200), anti-rabbit AF647 (1:200), anti-chicken AF647 (1:200) and anti-goat AF647 (1:200), hosted in donkey.

### Image processing

Images underwent processing and file-type conversion using ImageJ software (version 1.53t, National Institutes of Health, Bethesda, MD). Processed images were rendered in 3D using Dragonfly software (Version 2021.1 for [Windows]; Comet Technologies Canada Inc., Montreal, Canada). This software is available at https://www.theobjects.com/dragonfly.

### Statistical analysis

Quantitative data were analysed and the statistical tests detailed in the figure legends were carried out using Prism 8.0.2 (GraphPad). As viral titres are presented on a logarithmic scale and have exponential growth, the raw TCID50/ml values obtained from titration were log-transformed (Y = log[Y]) using GraphPad Prism prior to statistical analysis (as presented in Fig. 1A-C, Fig. 4A and Fig. S2) to aid in visualisation of the error bars and allow for better representation of the central point of data, as encouraged by Richardson et al.^42^. The mean titres detailed in the text have been back-transformed (Y = 10^y^) from this transformed data to align with the data presented in the figures. 2-way ANOVA with multiple comparison was employed to allow for comparison of all means of each different condition across time.

## Supplementary information

Supplementary Information

Supplementary video

## Data Availability

The datasets used and/or analysed during the current study are available from the corresponding author upon reasonable request.
