# Supplementary material for: Usutu virus and West Nile virus use a transcellular route of neuroinvasion across an in vitro model of the human blood–brain barrier
Source: Npj Viruses. 2024 Jul 25;2:32. doi: 10.1038/s44298-024-00034-4 (PMC11721115; doi:10.1038/s44298-024-00034-4)
Supplement: Supplementary file 1 — Supplementary Information [file 44298_2024_34_MOESM1_ESM.pdf]

Supplementary video 1. 3D rendering of the triple-cocultured *in vitro* blood-brain barrier (BBB) model layout with brain microvascular endothelial cells (BMECs) in apical compartment, astrocytes and pericytes in basolateral compartment and the transwell membrane separating the two. VE-cadherin shown in white. GFAP shown in red. PDGFR-β shown in green. Nuclei shown in blue. Dragonfly ORS used for rendering of confocal images.

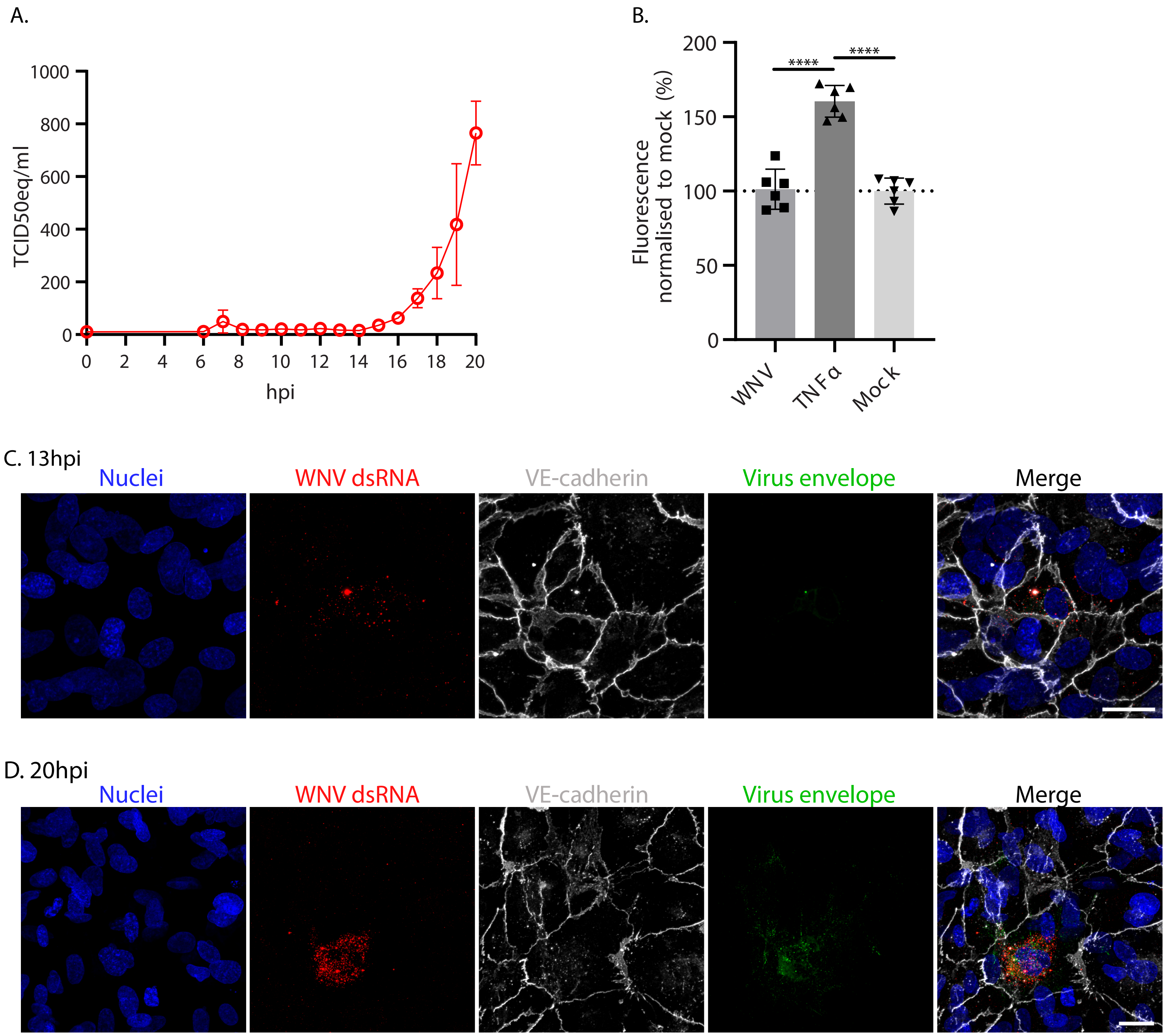

Supplementary figure 1. West Nile virus invades across the *in vitro* blood-brain barrier in absence of barrier disruption, after an initial round of replication in brain microvascular endothelial cells. A. TCID50 equivalents of West Nile virus (WNV) in the basolateral compartment of the *in vitro* blood-brain barrier (BBB) infected at a multiplicity of infection of 1. Two replicates were used per time-point, for a total of 32 *in vitro* BBBs across the course of the experiment. Representative data from 2 independent experiments. qPCR was used to quantify presence of viral genome in cell lysates which was compared against a standard curve of diluted virus stock to obtain TCID50eq values. Mean with SD. **B.** Leakage of a 20kDa fluorescent dextran through the *in vitro* BBB model at 24hpi with WNV at an MOI of 1 or stimulation with TNF-α, relative to mock infection. n=2. 3 replicates per condition, per experiment. Mean with SD. **** $p<0.0001$. One-way ANOVA. **C.** IF staining of WNV infected BMECs in apical compartment of *in vitro* BBB at 13hpi and **D.** 20hpi. Scale bars represent 50μm.

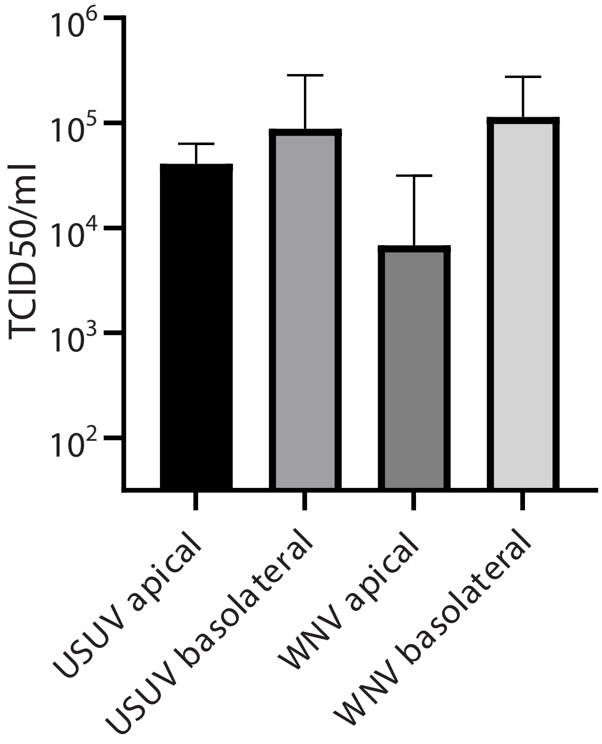

Supplementary figure 2. The transwell membrane does not prevent diffusion of virus into the basolateral compartment. Titres of Usutu virus and West Nile virus in apical and basolateral compartments of empty, coated transwells immediately following the inoculation incubation. n=1. 3 replicates per condition. Mean with SD. Data displayed has been log-transformed (Y=log[Y]).

Supplementary figure 3.

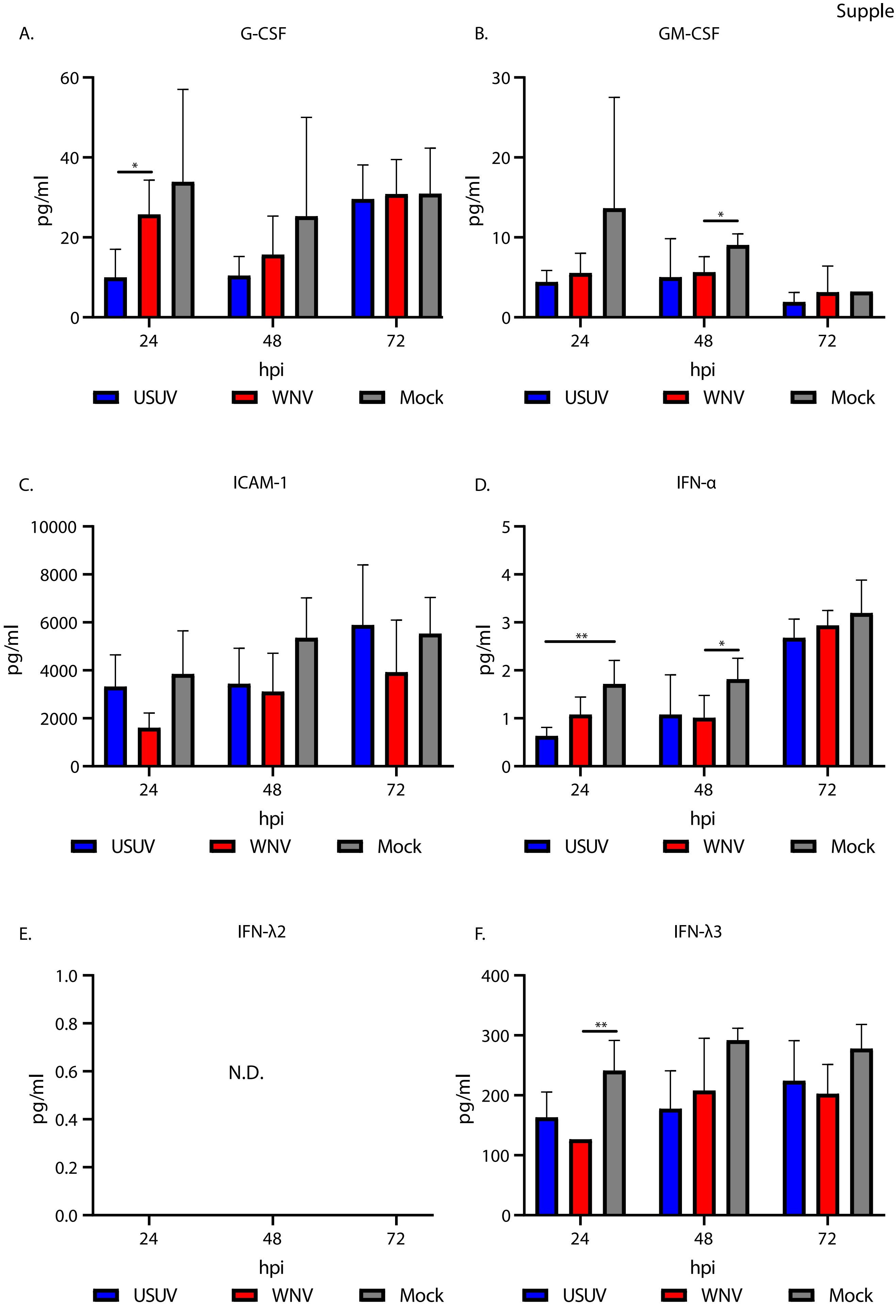

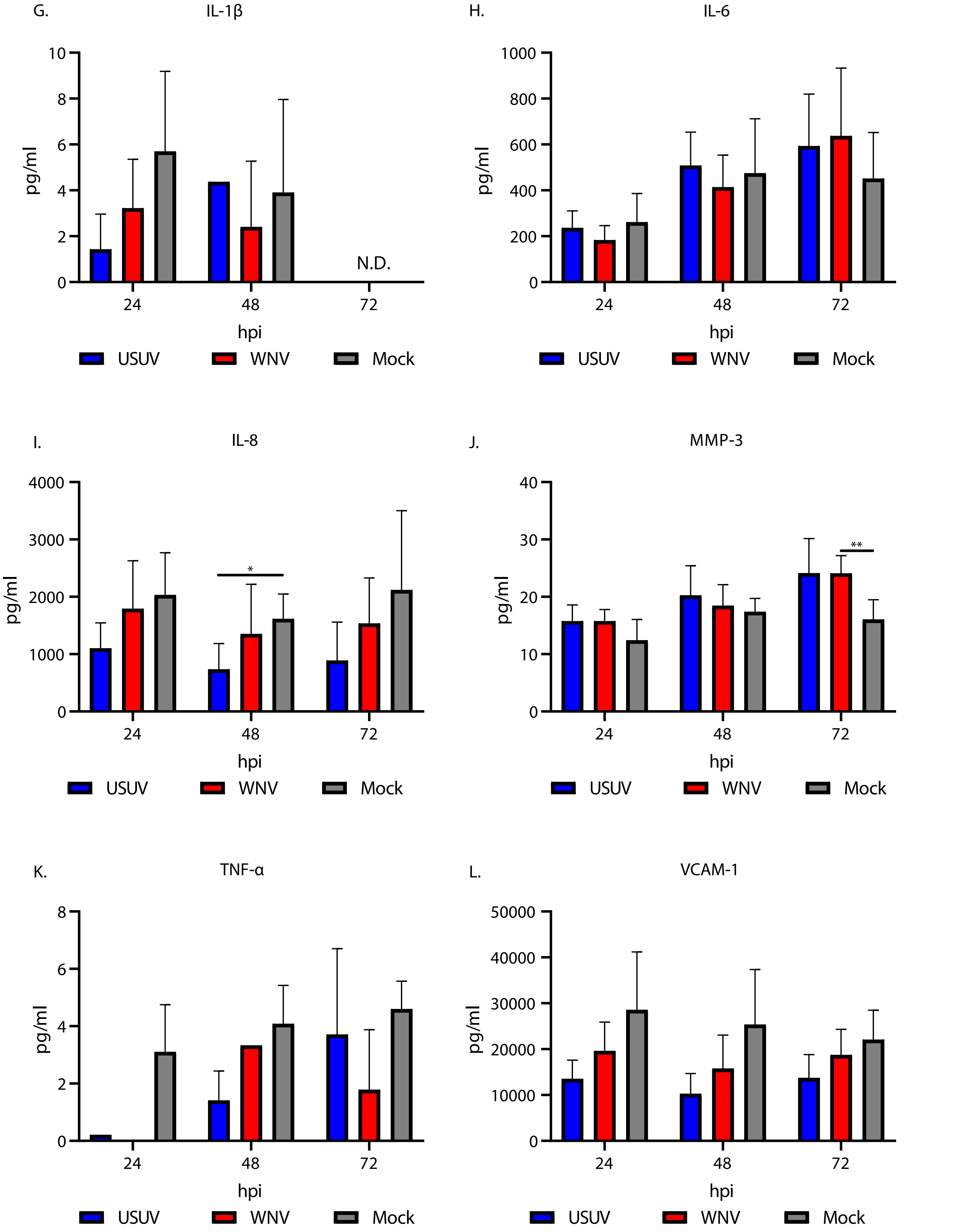

Supplementary figure 3. Concentration of **A.** G-CSF (* p=0.0214), **B.** GM-CSF (* p=0.0176), **C.** ICAM-1, **D.** IFN-α (* p=0.0295. ** p=0.0048), **E.** IFN-λ2, **F.** IFN-λ3 (** p=0.0057), **G.** IL-1β, **H.** IL-6, **I.** IL-8 (* p=0.0238), **J.** MMP-3 (** p=0.0043), **K.** TNF-α and **L.** VCAM-1 in basolateral supernatants from *in vitro* blood-brain barriers infected at a multiplicity of infection of 1 with West Nile virus (WNV) or Usutu virus (USUV). n=2. 3 replicates per condition, per experiment. Mean with SD. 2-way ANOVA with multiple comparison.
